# Amitriptyline for post-COVID headache: effectiveness, tolerability, and response predictors

**DOI:** 10.1007/s00415-022-11225-5

**Published:** 2022-07-12

**Authors:** Alicia Gonzalez-Martinez, Ángel Luis Guerrero-Peral, Susana Arias-Rivas, Lorenzo Silva, Álvaro Sierra, Ana Beatriz Gago-Veiga, David García-Azorín

**Affiliations:** 1 Headache Unit, Department of Neurology, Hospital Universitario de La Princesa and Instituto de Investigación Sanitaria Princesa (IIS Princesa), Madrid, Spain; 2 Department of Neurology, Universidad Autónoma de Madrid, Madrid, Spain; 3 Headache Unit, Department of Neurology, Hospital Clínico Universitario de Valladolid, Valladolid, Spain; 4 Department of Medicine, Universidad de Valladolid, Avda. Ramón y Cajal 3, 47005 Valladolid, Spain; 5 Department of Neurology, Hospital Santiago de Compostela, Galicia, Spain; 6 Department of Neurology, Hospital Universitario Puerta de Hierro, Madrid, Spain

**Keywords:** Migraine, Amitriptyline, COVID-19, Adverse events, Real-world evidence, Real-world effectiveness, Post-COVID-19 headache, Tension-type headache, Long-term effect, Long-haulers

## Abstract

**Background:**

Headache is one of the most frequently reported symptoms in post-COVID patients. The clinical phenotype of COVID-19 headache combines phenotypic features of both tension-type headache (TTH) and migraine. We aimed to assess the effectiveness, side effects and predictors of amitriptyline (AMT) response in a real-world study setting.

**Methods:**

We performed an observational multicentric study with a retrospective cohort. All consecutive patients with confirmed COVID-19 infection who received AMT for post-COVID headache from March 2020 to May 2021 were included. Response was evaluated by the reduction in the number of headache days per month (HDM) between weeks 8 and 12, compared with the baseline. We explored which variables were associated with a higher probability of response to AMT.

**Results:**

Forty-eight patients were eligible for the study, 40/48 (83.3%) females, aged 46.85 (SD: 13.59) years. Patients had history of migraine 15/48 (31.3%) or TTH 5/48 (10.4%). The mean reduction of HDM was 9.6 (SD: 10.9; 95% CI 6.5, 12.7) days. Only 2/48 (5%) of patients discontinued AMT due to poor tolerability. History of TTH (10.9, 95% CI 1.3, 20.6) and nausea (− 8.5, 95% CI − 14.6, − 2.5) were associated with AMT response.

**Conclusions:**

This study provides real-world evidence of the potential benefit of AMT in patients with post-COVID-19 headache, especially in patients with history of TTH and without concomitant nausea.

**Supplementary Information:**

The online version contains supplementary material available at 10.1007/s00415-022-11225-5.

## Background

Headache is one of the most frequent symptoms of severe acute respiratory syndrome coronavirus-2 (SARS-CoV-2) [1]. It is also commonly reported as a post-coronavirus disease (COVID-19) symptom, with a prevalence ranging from 8 to 15% during the first 6 months after SARS-CoV-2 infection [2, 3]. A study that followed 905 COVID-19 patients with headache for 9 months observed that patients who still had headache 2 months after the acute phase of the disease were likely to experience headache within the following months, being spontaneous improvement highly improbable [4].

Headache is a disabling symptom. Patients who suffer from headache during the acute phase describe it as the worst symptom [1, 5]. The clinical phenotype of COVID-19 headache combines phenotypic features of both tension-type headache (TTH) and migraine [6–9]; indeed, a study that analyzed whether patients fulfilled the International Classification of Headache Disorders (ICHD-3) [10] criteria for TTH or migraine observed that 54% patients fulfilled phenotypic criteria for TTH and 25% for migraine [9].

Amitriptyline (AMT) is a tricyclic antidepressant that has demonstrated efficacy in the treatment of both TTH [11, 12] and migraine [13]. It is one of the main prophylactic treatments recommended in the international guidelines for these conditions [14, 15], and frequently prescribed in routine clinical practice [16]. Moreover, potential benefits have been observed in the treatment of other comorbidities associated with COVID-19 such as insomnia, anxiety or other concomitant pain disorders make it an interesting option [2, 3].

In the present study, we aim to describe patients with post-COVID headache treated with AMT in clinical practice, describing tolerability and exploring the possible predictors of response. We hypothesized that patients with post-COVID-19 headache would respond to AMT.

## Method

### Study population and data collection

This is an observational analytical multicentric study with a retrospective cohort design.

The study was conducted according to the Strengthening the Reporting of Observational Studies in Epidemiology (STROBE) guidelines [17]. The study period encompassed March 2020–May 2021. Recruitment followed a non-probabilistic convenience sampling method, and every patient with post-COVID headache was assessed for eligibility. Patients were treated with AMT as the preventive treatment for post-COVID-19 headache as per physician in charge.

### Eligibility criteria

The study population was patients with post-COVID-19 headache treated with AMT. Post-COVID-19 headache was defined by headache starting during acute phase and lasting for more than 3 months, fulfilling ICHD-3 criteria for chronic headache attributed to systemic viral infection, not better accounted for by another ICHD-3 disorder [10]. The study settings were four third-level university hospitals from three major cities of Spain where headache diaries are collected on a regular basis.

The inclusion criteria were: (1) Confirmed COVID-19 disease, either by polymerase chain reaction (PCR) or serum antibody testing, (2) headache during the acute phase of COVID-19, (3) persistent headache after the resolution of the acute symptoms for at least 3 months, (4) age over 18 years, and (5) minimum follow-up of 12 weeks. Exclusion criteria: (1) incompleteness of data, (2) death during follow-up.

### Study endpoints

The primary endpoint was the change in the number of headache days between the baseline, defined as the 4 weeks preceding AMT use, and weeks 8–12 during AMT use. As secondary endpoints we included 30% (partial), 50% (standard) and 75% (optimal) response rates, defined as proportion of patients with 30%, 50%, 75% reduction in monthly days with headache frequency from baseline to weeks 8–12. We evaluated the change in the number of intense headache days-defined as headache intensity higher or equal than 7 in a 0–10 Numerical Rating Scale (NRS)-, and the change of acute medication days between the baseline and weeks 8–12 during AMT use. As exploratory endpoints, we also assessed which predictive variables associated with a higher reduction in headache days.

### Variables included in the study

A complete medical history was obtained from each patient during an in-person clinical interview, done by a neurologist with experience in headache disorders. The demographic and clinical variables included sex, age, history of migraine and TTH, history of medication overuse (MOH), anxiety or depression, insomnia and other concomitant pain. Presence of comorbid psychiatric disorders, including anxiety or depression, was evaluated at the time when amitriptyline was used Headache specific variables included prior history of headache, and the specific headache type, prior number of prophylactics for post-COVID-19 headache if any, as well as headache phenotype (intensity measured by NRS between 0 (no pain) and 10 (worst possible pain), localization (hemicranial, holocranial), quality of pain (oppressive, throbbing), headache accompanying symptoms (nausea, vomiting, photophobia, phonophobia, osmophobia, allodynia, worsening with physical activity), headache variables (baseline number of headache days per month, baseline number of moderate–severe headache days per month defined as 4–10 in NRS, baseline urgent care visits per month, baseline number of acute medication days per month), COVID-19 related variables (time elapsed from COVID-19 to AMT onset) and AMT use (AMT starting dose, maximum AMT dose, time lapsed before doubling the starting dose, AMT duration, AMT dose at adverse effects, NSAIDs and triptans as symptomatic treatment). All the variables included in the study are available in Supplementary Table 1.

### Statistical analysis

We present categorical variables as frequencies and percentages and continuous variables as means and standard deviations (SD) or medians and interquartile ranges (IQR) if the distribution was not normal was determined by the Kolmogorov–Smirnov test and homogeneity of variance using Levene test. We used the Chi-square or Fisher’s exact test to compare qualitative variables between responders and non-responders and paired Student’s *t* test or Mann–Whitney *U* test depending on the distribution of the quantitative variables. In the comparison of the clinical situation between the baseline and weeks 8–12, paired *T* test and Wilcoxon tests were used accordingly. All endpoints were evaluated on an intention-to-treat basis. For response predictors’ evaluation, we used a univariate linear regression analysis, assessing which variables were associated with a higher reduction in the number of headache days; variables with a *p* value below 0.1 were included in a multivariable regression analysis. Moreover, we performed a comparison between responders and non-responders by weeks 8–12. In all comparisons, tests were two-tailed, being accepted the statistical significance if the *p* value was < 0.05. We present odds ratios (OR) with 95% confidence intervals (CI). To address missing data, a conservative imputation technique was used (last observation carried forward) for variables with variation over time (e.g., headache days per month), and in the case of non-evolutionary variables, complete case analysis was used. The statistical analyses were done using SPSS v26.0 (IBM Corp. Armonk, NY). Sample size was based on available data. We performed primary analysis of these data and no other analyses of these data have been published.

### Ethics approval and consent to participate

Written informed consent was waived by ethics committee CEIm Área de Salud Valladolid Este (PI: 21 2280), due to the study being a review of anonymized medical records. The study was done according to the principles of the Declaration of Helsinki [18].

## Results

During the study period, 48 patients fulfilled eligibility criteria. Patients were female in 40/48 (83.3%) cases. Among patients with prior history of migraine or TTH, 12/20 (60%) of patients had ever received a prophylactic treatment, and median number of prophylactic treatments was 1 (IQR: 0–1). Regarding AMT, 6/20 (30%) had previously received it as prophylactic treatment for their primary headache, and therefore, 6/48 (12.5%) had been exposed to AMT before COVID-19 headache. At the time of AMT initiation, the mean age of patients was 46.8 (SD: 13.6). History of migraine was present in 15/48 (31.3%), history of TTH in 5/48 (10.4%) and prior history of medication-overuse headache (MOH) in 1/48 (2.1%). Among other comorbidities anxiety or depression were present in 11/48 (22.9%) of cases, prior history of insomnia in 11/48 (22.9%) and other concomitant pain syndromes in 7/48 (14.6%) patients. The median number of prior prophylactic treatments for post-COVID-19 headache was 0 (0–1). All the demographic variables are listed in Table 1.

**Table 1:** Demographic and clinical variables of the patients included in the study

Variable	All patients (*N* = 48)	Response (change in the number of headache days)		
		*B*	95% CI lower limit, CI upper limit	*p* value
Female, *n*/*N* (%)	40/48 (83.3%)	− 0.400	(− 9.047, 8.247)	0.926
Age, years				
*n*/*N* (%)	48/48 (100%)	− 0.051	(− 0.290, 0.189)	0.672
Mean (SD)	46.85 (13.59)			
Prior history of migraine	15/48 (31.3%)	1.842	(− 5.089, 8.774)	0.595
History of TTH	5/48 (10.4%)	11.535	(1.555, 21.515)	0.024*
Comorbid anxiety or depression	11/48 (22.9%)	7.862	(0.558, 15.167)	0.035*
Comorbid insomnia	11/48 (22.9%)	7.037	(− 0.342, 14.415)	0.061
Other concomitant pain	7/48 (14.6%)	2.063	(− 7.049, 11.174)	0.651
Prior number of prophylactics for post-COVID-19 headache				
*n*/*N* (%)	48/48 (100%)	− 0.268	(− 5.271, 4.735)	0.915
Median (Q1–Q3)	0 (0–1)			

*TTH* tension-type headache, *MOH* medication-overuse headache, *CI* confidence interval, *SD* standard deviation

^a^Std. error difference, 95% CI lower limit, CI upper limit

*p < 0.05

^#^*U* Mann–Whitney; NaN (not a number)

Post-COVID-19 headache had a median pain intensity of 7 [IQR: 5.2–9] in a NRS, 38/48 (79%) holocranial location, 44/48 (91%) oppressive quality and 12/48 (25%) throbbing quality. Accompanying symptoms included nausea 16/48 (33.3%), vomiting 6/48 (12.5%), photophobia and phonophobia 16/48 (33.3%), osmophobia 3/48 (6.3%). Variables related with post-COVID-19 headache and AMT are included in Tables 2 and 3.

**Table 2:** COVID-19-associated headache phenotype regarding ATM response

COVID-19 headache characteristics	All patients (*N* = 48)	Response (change in the number of headache days)			
		*B*	95% CI lower limit, CI upper limit		*p* value
Intensity (VAS)					
*n*/*N* (%)	48/48 (100%)	0.243		(− 1.296, 1.782)	0.752
Median (Q1–Q3)	7 (5.25–9)				
Hemicranial, *n*/*N* (%)	10/48 (20.8%)	− 4.632		(− 12.448, 3.185)	0.239
Holocranial, *n*/*N* (%)	38/48 (79.2%)	1.474		(− 6.450, 9.398)	0.710
Oppressive, *n*/*N* (%)	44/48 (91.7%)	1.545		(− 10.107, 13.198)	0.791
Throbbing, *n*/*N* (%)	12/48 (25%)	− 4.444		(− 11.770, 2.881)	0.228
Nausea, *n*/*N* (%)	16/48 (33.3%)	− 9.531		(− 15.756, − 3.307)	0.003*
Vomiting, *n*/*N* (%)	6/48 (12.5%)	− 6.286		(− 15.851, 3.279)	0.192
Photophobia, *n*/*N* (%)	16/48 (33.3%)	− 1.938		(− 8.750, 4.875)	0.570
Phonophobia, *n*/*N* (%)	16/48 (33.3%)	− 4.469		(− 11,176, 2.238)	0.186
Osmophobia, *n*/*N* (%)	3/48 (6.3%)	− 10.493		(− 23.683, 2.698)	0.116
Allodynia, *n*/*N* (%)	6/48 (12.5%)	− 2.993		(− 12.658, 6.673)	0.536
Worsening with headache movement, *n*/*N* (%)	28/48 (58.3%)	− 2.447		(− 8.906, 4.012)	0.449

VAS Visual Analogue Scale

^a^Std. error difference

*p < 0.05

^#^Mann–Whitney *U*

**Table 3:** Variables associated with post-COVID-19 headache treated with AMT

Variables	All patients (*N* = 48)	Response (change in the number of headache days)		
		*B*	95% CI lower limit, CI upper limit	*p* value
Baseline number of headaches per month (days), median (Q1–Q3)	30 (30–30)	0.352	(− 0.173, 0.878)	0.183
Baseline number of moderate-severe headache days per month (days), median (Q1–Q3)	10 (4–20)	0.154	(− 0.183, 0.491)	0.363
Baseline number of NSAIDs per month (days), median (Q1–Q3)	12(5.25–23.75)	0.104	(− 0.210, 0.418)	0.510
Baseline number of triptans per month (days), median (Q1–Q3)	0 (0–0)	− 1.119	(− 3.173, − 3.173)	0.278
Time from COVID-19 to Amitriptyline (months)		− 0.641	(− 1.402, − 0.042)	0.036*
*n*/*N* (%)	48/48 (100%)			
Median (Q1–Q3)	4 (2–7)			
Amitriptyline starting dose (mg), median (Q1–Q3)	10 (10–10)	− 0.633	(− 1.257, − 0.009)	0.047*
Amitriptyline maximum doses (mg), median (Q1–Q3)	25 (12.5–25)	− 0.069	(− 0.349, 0.210)	0.621
Time lapsed before doubling the starting dose (weeks)	2 (0–4)	0.309	(− 0.287, 0.906)	0.302
Amitriptyline duration (months), median (Q1–Q3)	3(2–4)	0.292	(− 0.845, 1.429)	0.607
NSAIDs as symptomatic treatment, *n*/*N* (%)	34/48 (70.8%)	− 1.681	(− 8.754, 5.393)	0.635
Triptan as symptomatic treatment, *n*/*N* (%)	4/48 (8.3%)	− 9.182	(− 20.520, 2.156)	0.110
Adverse effects, *n*/*N* (%)	19/43 (44.18%)	− 4.340	(− 11.147, 2.467)	0.205

*NSAIDs* non-steroidal anti-inflammatory drugs

^a^Std. error difference

*p < 0.05

^#^Mann–Whitney *U*

In the month prior to AMT use, the median number of headache days per month was 30 [IQR: 30–30, range 7–30], and the median number of moderate-intense headache days per month was 10 (4–20). The median number of preventive treatments used for post-COVID-19 headache prior to AMT was 0 [IQR: 0–1]. The median number of days of non-steroidal anti-inflammatory drugs (NSAIDs) as acute medication was 12 [IQR: 5–24] and the median number of triptans per month was 0 [IQR: 0–0, range 0–10] per month. Time from COVID-19 to AMT was 4 [IQR: 2–7] months.

### Amitriptyline use

**Fig. 1:**
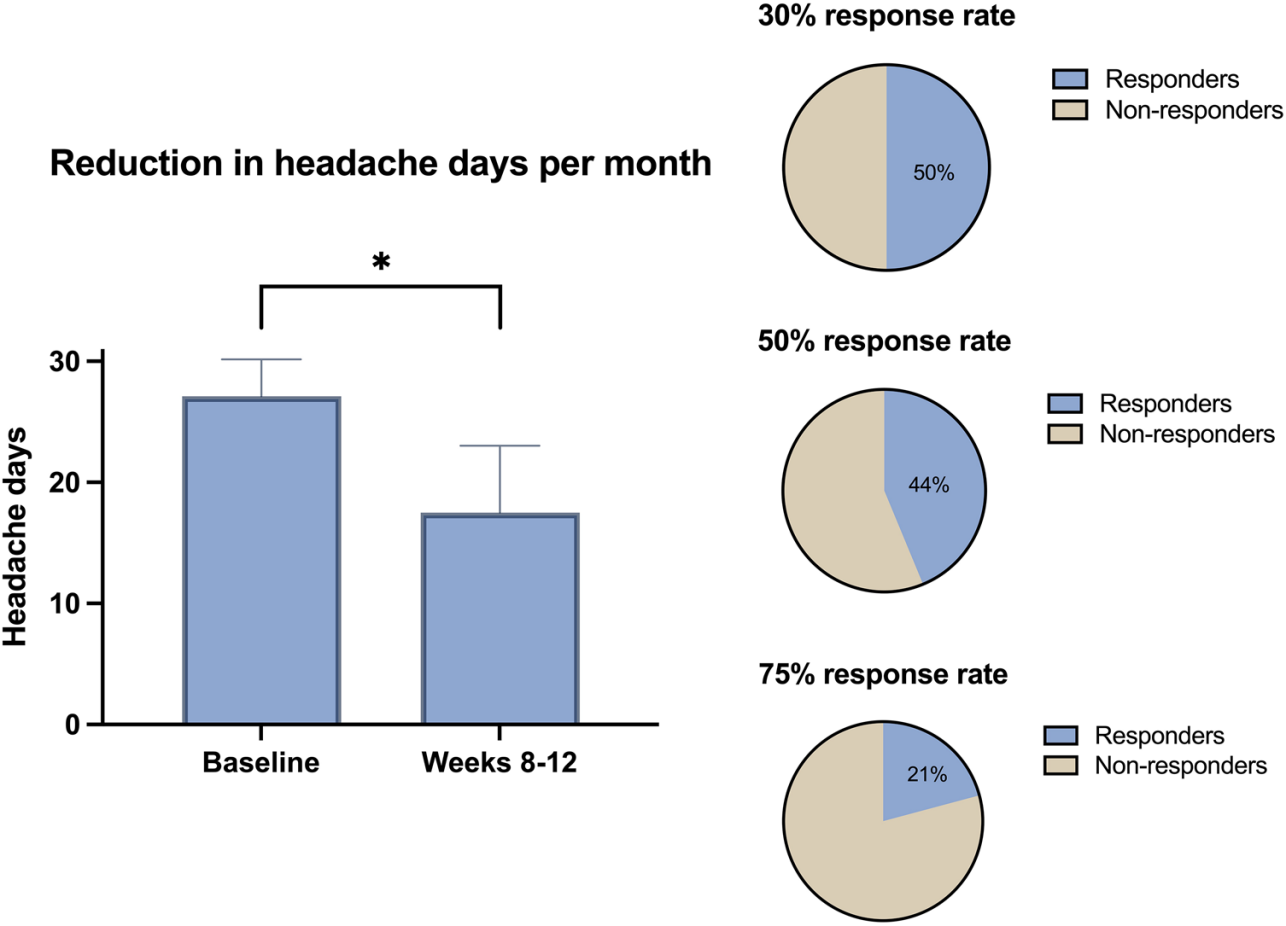
Changes in headache days per month at baseline and between weeks 8 and 12 during AMT use. *AMT* amitriptyline. *p < 0.05

The starting dose was 10 mg in 40/48 (83.3%) patients. The maximum achieved dose of AMT was 25 mg in 26/48 (54.2%) patients, 50 mg 2/48 (4.2%) patients and 75 mg in 1/48 (2.1%) patients. The time lapsed before doubling the starting dose was 1 week in 8/48 (16.7%) patients, 2 weeks in 8/48 (16.7%) patients and 4 weeks in 9/48 (18.8%). AMT main starting dose and maximum dose are summarized in Fig. 1S. Three patients discontinued AMT due to adverse effects, with a retention rate of 95%. Adverse events are listed in Supplementary Table 2.

### Primary endpoint: change in the number of headache days

There was a 9.6 (SD: 10.9; CI 6.5, 12.7) headache days’ reduction between the baseline and weeks 8–12 during AMT use (*p* < 0.001). Figure 1 summarizes the changes in headache days per month at baseline and between weeks 8 and 12.

### Secondary endpoints: response rate, intense headache days and acute medication days

The proportion of patients with a 30% response by weeks 8–12 was 24/48 (50%), 50% response by weeks 8–12 was 21/48 (43.7%) and 75% response by weeks 8–12 was 10/48 (21%) (Fig. 1). There was a statistically significant reduction in the number of intense headache days per month and acute medication days, when comparing weeks 8–12 and the baseline (all *p* < 0.001).

### Exploratory endpoints: predictors of amitriptyline response

In the univariate linear regression analysis, time from COVID-19 to AMT onset, history of TTH, anxiety or depression, nausea and AMT starting dose were associated with a reduction in the number of headache days in Table 3 (Supplementary Table 1) and two variables, prior history of TTH (B value 10.9, 95% CI 1.3, 20.6, *p* value: 0.024) and nausea (*B* value − 8.5, 95% CI − 14.6, − 2.5), *p* value: 0.007) remained statistically significant in the multivariable regression analysis (Supplementary Table 1). Table 4 includes the full details of the multivariable linear regression.

**Table 4:** Univariate and multivariate regression analysis

Variable	Analysis	*B* value	95% CI lower limit, CI upper limit	*p* value
Months from COVID-19 to AMT	Univariate	− 0.641	(− 1.402, − 0.042)	0.036*
	Multivariate	− 0.429	(− 1.253, 0.395)	0.299
History of TTH	Univariate	11.535	(1.555, 21.515)	0.024*
	Multivariate	10.966	(1.316, 20.617)	0.027*
Comorbid anxiety or depression	Univariate	7.862	(0.558, 15.167)	0.035*
	Multivariate	2.778	(− 4.661, 10.216)	0.455
Comorbid insomnia	Univariate	7.037	(− 0.342, 14.415)	0.061
	Multivariate	1.687	(− 5.916, 9.290)	0.656
Nausea	Univariate	− 9.531	(− 15.756, − 3.307)	0.003*
	Multivariate	− 8.547	(− 14.624, − 2.470)	0.007**
Initial dose of Amitriptyline (mg)	Univariate	− 0.633	(− 1.257, − 0.009)	0.047*
	Multivariate	− 0.322	(− 0.909, 0.265)	0.275

*TTH* tension-type headache, *CI* confidence interval

*p < 0.05

**p < 0.01

## Discussion

In the present study, we assessed the real-world effectiveness of AMT for the treatment of post-COVID-19 headache. We observed a 9-day median reduction of headache days per month 3 months after the treatment initiation. Half of the patients had a partial (30%) response, 44% a standard (50%) response and 21% an optimal (75%) response.

One of the main limitations of the study was the absence of a control group. The observed effect of AMT could be masked by the natural history of post-COVID-19 headache, in which some patients present spontaneous improvement over time [19]. However, post-COVID-19 patients that still suffer from headache 2 months after the acute phase are likely to persist with headache after 9 months, according to a multicentric study that included 905 patients with post-COVID-19 headache [4]. For this reason, we will contextualize our results with that of the randomized control studies (RCTs) of AMT in other headache disorders, as migraine and TTH.

Five RCTs support the benefit of AMT in patients with TTH [20–24]. In the studies that describe the reduction in headache days at 8–12 weeks, the mean reduction in headache days ranges from 1.5 to 6 days [21, 22]. Studies reporting the 50% response rate at 8–12 weeks describe it in 36% of patients [20]. In the case of migraine, three RCTs comparing AMT to placebo support the benefit of AMT as a prophylactic treatment for migraine [25–28]. In the studies that describe the reduction in migraine headache days per month at 8–12 weeks, there is a reduction of up to five migraine headache days [28]. Studies reporting the proportion of patients with a 50% response rate at weeks 8–12 describe it in 55% of patients [25].

An interesting finding of our study was the higher probability of response in patients with history of TTH. This could be related with the phenotypic presentation of post-COVID-19 headache, which in half of the patients fulfills the phenotypic ICHD-3 criteria for TTH [9], and in many cases combines features of both TTH and migraine.

Moreover, AMT was originally developed as an antidepressant, with the first studies published back in 1960’s. It is frequently used in the treatment of anxiety, insomnia and other pain disorders, which makes it a useful option in patients with these comorbidities [27, 29–32], being many of these reported by post-COVID-19 patients [33]. In our sample, 22% had anxiety or depression, 22% insomnia and 14% had other concomitant pain as comorbidities; however, the presence of these was associated with a higher probability of response.

Oral prophylactics have been associated with tolerability problems, which may lead to treatment discontinuation. In the case of AMT, it may cause somnolence, cognitive disturbances and dry mouth, among others [34]. In the TTH and migraine RCTs, the proportion of patients that experienced treatment-related adverse effects (TRAE) was between 28 and 97%; nevertheless, only 7–9% of patients discontinued the treatment due to tolerability reasons [20–22, 25], in line with our findings. The smaller proportion of TRAE and discontinuation may be explained by the relatively low doses that were used as starting dose in most of the cases, since several AEs are dose-dependent [35], facilitating a significant reduction in monthly headache days in post-COVID-19 headache. A progressive titration of the drug is generally recommended, together with monitoring the clinical response and tolerability. AMT seems to be a reasonable treatment for post-COVID-19-associated headache in terms of a risk–benefit ratio at low doses.

Our study has relevant limitations. It is a retrospective study with a small sample size, and therefore, some differences might not be addressed in the current series, and other psychiatric comorbidities were not evaluated. Moreover, as it has been previously discussed, the lack of a placebo-controlled comparison group may have overvalued the positive effect seen in this study. An additional limitation would be the intrinsic selection bias typical of a tertiary referral headache center. Larger scale, randomized placebo-controlled studies would be desirable to evaluate the intrinsic effect of AMT in this group of patients as well as the long-term effectiveness of AMT beyond 12 weeks and the sustained effect of the treatment over time.

## Conclusions

This study provides class-4 real-world evidence of possible benefit of AMT in the treatment of post-COVID-19 headache. There was a statistically significant reduction in the number of headache days 3 months after AMT use, with a median change was almost 10 headache days per month less, The proportion of patients with a 50% response was 44%, and 20% of patients had an optimal response. Moreover, there was a significant reduction in the number of intense headache days per month and acute medication days 3 months after AMT use. History of TTH, anxiety or depression, absence of nausea, time from COVID-19 to AMT and amitriptyline starting dose in the univariate analysis, and TTH and the absence of nausea were associated with a higher probability of response. Treatment discontinuation due to adverse effects was infrequent.

## Supplementary Information

Below is the link to the electronic supplementary material.

Supplementary file1 (DOCX 39 KB)

Supplementary file2 (JPG 672 KB)

Supplementary file3 (DOCX 21 KB)

## Data Availability

Anonymized data will be shared by request from any qualified investigator.

## Abbreviations

TTH: Tension-type headache
MOH: Medication overuse headache
STROBE: Strengthening the reporting of observational studies in epidemiology
ICHD: International classification of headache disorders
SD: Standard deviation
IQR: Inter-quartile range
OR: Odds ratio
CI: Confidence interval
