# Supplementary material for: Amitriptyline for post-COVID headache: effectiveness, tolerability, and response predictors
Source: J Neurol. 2022 Jul 12;269(11):5702–9. doi: 10.1007/s00415-022-11225-5 (PMC9553757; doi:10.1007/s00415-022-11225-5)
Supplement: Supplementary file 1 — Supplementary file1 (DOCX 39 KB) [file 415_2022_11225_MOESM1_ESM.docx]

STROBE Statement—checklist of items that should be included in reports of observational studies

|  | Item No. | Recommendation | Page  No. | Relevant text from manuscript |
| --- | --- | --- | --- | --- |
| **Title and abstract** | 1 | (*a*) Indicate the study’s design with a commonly used term in the title or the abstract | 1&2 | “retrospective cohort design” |
|  |  | (*b*) Provide in the abstract an informative and balanced summary of what was done and what was found | 1&2 | “retrospective cohort design” |
| Introduction | | | |  |
| Background/rationale | 2 | Explain the scientific background and rationale for the investigation being reported | 4&5 | “potential benefits have been observed in the treatment of other comorbidities associated with COVID-19 such as insomnia, anxiety or other concomitant pain disorders” |
| Objectives | 3 | State specific objectives, including any prespecified hypotheses | 4&5 | “In the present study we aim to describe the clinical outcome of patients with persistent post-COVID headache treated with AMT in clinical practice, describing tolerability and exploring the possible predictors of response.” |
| Methods | | | |  |
| Study design | 4 | Present key elements of study design early in the paper | 5&6 | “This is an observational analytical multicentric study with a retrospective cohort design.” |
| Setting | 5 | Describe the setting, locations, and relevant dates, including periods of recruitment, exposure, follow-up, and data collection | 5&6 | “The study period encompassed March 2020-May 2021. Recruitment.” |
| Participants | 6 | (*a*) *Cohort study*—Give the eligibility criteria, and the sources and methods of selection of participants. Describe methods of follow-up  *Case-control study*—Give the eligibility criteria, and the sources and methods of case ascertainment and control selection. Give the rationale for the choice of cases and controls  *Cross-sectional study*—Give the eligibility criteria, and the sources and methods of selection of participants | 5 | “Recruitment followed a non-probabilistic convenience sampling method, and every patient with post-COVID headache was assessed for eligibility.” “The inclusion criteria were: 1) Confirmed COVID-19 disease, either by polymerase chain reaction (PCR) or serum antibody testing, 2) headache during the acute phase of COVID-19, 3) persistent headache after the resolution of the acute symptoms for at least 3 months, 4) age over 18 years old, 5) minimum follow-up of 12 weeks. Exclusion criteria: 1) incompleteness of data, 2) death during follow-up.” |
|  |  | (*b*) *Cohort study*—For matched studies, give matching criteria and number of exposed and unexposed  *Case-control study*—For matched studies, give matching criteria and the number of controls per case | 5 |  |
| Variables | 7 | Clearly define all outcomes, exposures, predictors, potential confounders, and effect modifiers. Give diagnostic criteria, if applicable | 6&7 | “The primary endpoint was the change in the number of headache days between the baseline, defined as the 4 weeks preceding AMT use, and weeks 8-12 after AMT use.” |
| Data sources/ measurement | 8* | For each variable of interest, give sources of data and details of methods of assessment (measurement). Describe comparability of assessment methods if there is more than one group | 6&7 | “We evaluated the change in the number of intense headache days (defined as headache intensity higher or equal than 7 in a 0-10 numerical rating scale (NRS), and the change of acute medication days between the baseline and weeks 8-12 after AMT use.” |
| Bias | 9 | Describe any efforts to address potential sources of bias | 6&7 | “To address missing data, a conservative imputation technique was used (last observation carried forward) for variables with variation over time (e.g. headache days per month) and in the case of non-evolutionary variables, complete case analysis was used.” |
| Study size | 10 | Explain how the study size was arrived at | 6,7&8 | “The study period encompassed March 2020-May 2021. Recruitment followed a non-probabilistic convenience sampling method, and every patient with post-COVID headache was assessed for eligibility.” |

Continued on next page

| Quantitative variables | 11 | Explain how quantitative variables were handled in the analyses. If applicable, describe which groupings were chosen and why | 6,7&8 | “We used the chi-square or Fisher’s exact test to compare qualitative variables between responders and non-responders and Student’s t-test or Mann Whitney-U test depending on the distribution of the quantitative variables.” | | | | |
| --- | --- | --- | --- | --- | --- | --- | --- | --- |
| Statistical methods | 12 | (*a*) Describe all statistical methods, including those used to control for confounding | 7&8 | | | “For response predictors evaluation, we used a univariate linear regression analysis, assessing which variables were associated with a higher reduction in the number of headache days; variables with a p value below 0.1 were included in a multivariate regression analysis.” | | |
|  |  | (*b*) Describe any methods used to examine subgroups and interactions | 7&8 | | “Moreover, we performed a comparison between responders and non-responders by weeks 8-12. In all comparisons, tests were two-tailed, being accepted the statistical significance if the *P* value was <0.05. | | |  |
|  |  | (*c*) Explain how missing data were addressed | 7&8 | | To address missing data, a conservative imputation technique was used (last observation carried forward) for variables with variation over time (e.g. headache days per month) and in the case of non-evolutionary variables, complete case analysis was used. | | |  |
|  |  | (*d*) *Cohort study*—If applicable, explain how loss to follow-up was addressed  *Case-control study*—If applicable, explain how matching of cases and controls was addressed  *Cross-sectional study*—If applicable, describe analytical methods taking account of sampling strategy | 8 | | | | “5) minimum follow-up of 12 weeks. Exclusion criteria: 1) incompleteness of data, 2) death during follow-up.” |  |
|  |  | (*e*) Describe any sensitivity analyses | 8 | | | | N/A |  |
| Results | | | | | | | |  |
| Participants | 13* | (a) Report numbers of individuals at each stage of study—eg numbers potentially eligible, examined for eligibility, confirmed eligible, included in the study, completing follow-up, and analysed | 8,9&10 | | | | “During the study period, 48 patients fulfilled eligibility criteria. Patients were female in 40/48 (83.3%) cases.” |  |
|  |  | (b) Give reasons for non-participation at each stage | 8,9&10 | | | | “Three patients discontinued AMT due to adverse effects, with a retention rate of 95%. Adverse events are listed in supplementary Table 2.” |  |
|  |  | (c) Consider use of a flow diagram | 8,9&10 | | | | N/A |  |
| Descriptive data | 14* | (a) Give characteristics of study participants (eg demographic, clinical, social) and information on exposures and potential confounders | 8&9 | | | | “At the time of AMT initiation, the mean age of patients was 46.85 (SD: 13.59). Prior history of migraine was present in 15/48 (31.3%), prior history of TTH in 5/48 (10.4%) and prior history of medication-overuse headache (MOH) in 1/48 (2.1%). Among other comorbidities anxiety or depression were present in 11/48 (22.9%) of cases, prior history of insomnia in 11/48 (22.9%) and other concomitant pain syndromes in 7/48 (14.6%) patients. |  |
|  |  | (b) Indicate number of participants with missing data for each variable of interest | 8,9&10 | | | | There was a 9.6 (SD:10.97; CI: 6.50,12.70) headache days reduction between the baseline and weeks 8-12 after AMT use (p<0.001). Figure 1 summarizes the changes in headache days per month at baseline and between weeks 8-12. |  |
|  |  | (c) *Cohort study*—Summarise follow-up time (eg, average and total amount) | 8,9&10 | | | | “5) minimum follow-up of 12 weeks.” |  |
| Outcome data | 15* | *Cohort study*—Report numbers of outcome events or summary measures over time | *8,9&10* | | | | *N/A* |  |
|  |  | *Case-control study—*Report numbers in each exposure category, or summary measures of exposure | *NA* | | | | *N/A* |  |
|  |  | *Cross-sectional study—*Report numbers of outcome events or summary measures | *NA* | | | | *N/A* |  |
| Main results | 16 | (*a*) Give unadjusted estimates and, if applicable, confounder-adjusted estimates and their precision (eg, 95% confidence interval). Make clear which confounders were adjusted for and why they were included | 8,9&10, Tables 1-4, Table1S, Table 2S. | | | | “There was a 9.6 (SD:10.97; CI: 6.50,12.70) headache days reduction between the baseline and weeks 8-12 after AMT use (p<0.001).” |  |
|  |  | (*b*) Report category boundaries when continuous variables were categorized | 8,9&10, Tables | | | | N/A |  |
|  |  | (*c*) If relevant, consider translating estimates of relative risk into absolute risk for a meaningful time period | 8,9&10,Tables | | | | ” TTH (B value 10.966, 95% CI: 1.316, 20.617, p value: 0.024) and nausea (B value -8.547, 95% CI: -14.624, - -2.470), p value: 0.007) remained statistically significant in the multivariate regression analysis.” |  |

Continued on next page

| Other analyses | 17 | Report other analyses done—eg analyses of subgroups and interactions, and sensitivity analyses | 8,9&10, Tables 1-4, Table1S, Table 2S | In the univariate linear regression analysis, time from COVID-19 to AMT onset, prior history of TTH, prior history of anxiety or depression, nausea and AMT starting dose were associated with a reduction in the number of headache days in (Table 1S) and two variables, prior history of TTH (B value 10.966, 95% CI: 1.316, 20.617, p value: 0.024) and nausea (B value -8.547, 95% CI: -14.624, - -2.470), p value: 0.007) remained statistically significant in the multivariate regression analysis. |
| --- | --- | --- | --- | --- |
| Discussion | | | | |
| Key results | 18 | Summarise key results with reference to study objectives | 12&13 | “In the present study we assessed the real-world effectiveness of AMT for the treatment of post-COVID-19 headache. We observed a 9-days median reduction of headache days per month three months after the treatment initiation. Half of the patients had a partial response, 44% a standard response and 21% an optimal response.” |
| Limitations | 19 | Discuss limitations of the study, taking into account sources of potential bias or imprecision. Discuss both direction and magnitude of any potential bias | 10,11,12&13 | “An additional limitation would be the intrinsic selection bias typical of a tertiary referral headache center. |
| Interpretation | 20 | Give a cautious overall interpretation of results considering objectives, limitations, multiplicity of analyses, results from similar studies, and other relevant evidence | 12&13 | “Moreover, as it has been previously discussed, the lack of a placebo-controlled comparison group may have overvalued the positive effect seen in this study.” |
| Generalisability | 21 | Discuss the generalisability (external validity) of the study results | 12&13 | “Our study has relevant limitations. It is a retrospective study with a small sample size and therefore some differences might not be addressed in the current series.” |
| Other information | |  | | |
| Funding | 22 | Give the source of funding and the role of the funders for the present study and, if applicable, for the original study on which the present article is based | 13 | N/A |

*Give information separately for cases and controls in case-control studies and, if applicable, for exposed and unexposed groups in cohort and cross-sectional studies.

**Note:** An Explanation and Elaboration article discusses each checklist item and gives methodological background and published examples of transparent reporting. The STROBE checklist is best used in conjunction with this article (freely available on the Web sites of PLoS Medicine at http://www.plosmedicine.org/, Annals of Internal Medicine at http://www.annals.org/, and Epidemiology at http://www.epidem.com/). Information on the STROBE Initiative is available at www.strobe-statement.org.
