# Supplementary figures and images for: Amitriptyline for post-COVID headache: effectiveness, tolerability, and response predictors
Source: J Neurol. 2022 Jul 12;269(11):5702–9. doi: 10.1007/s00415-022-11225-5 (PMC9553757; doi:10.1007/s00415-022-11225-5)

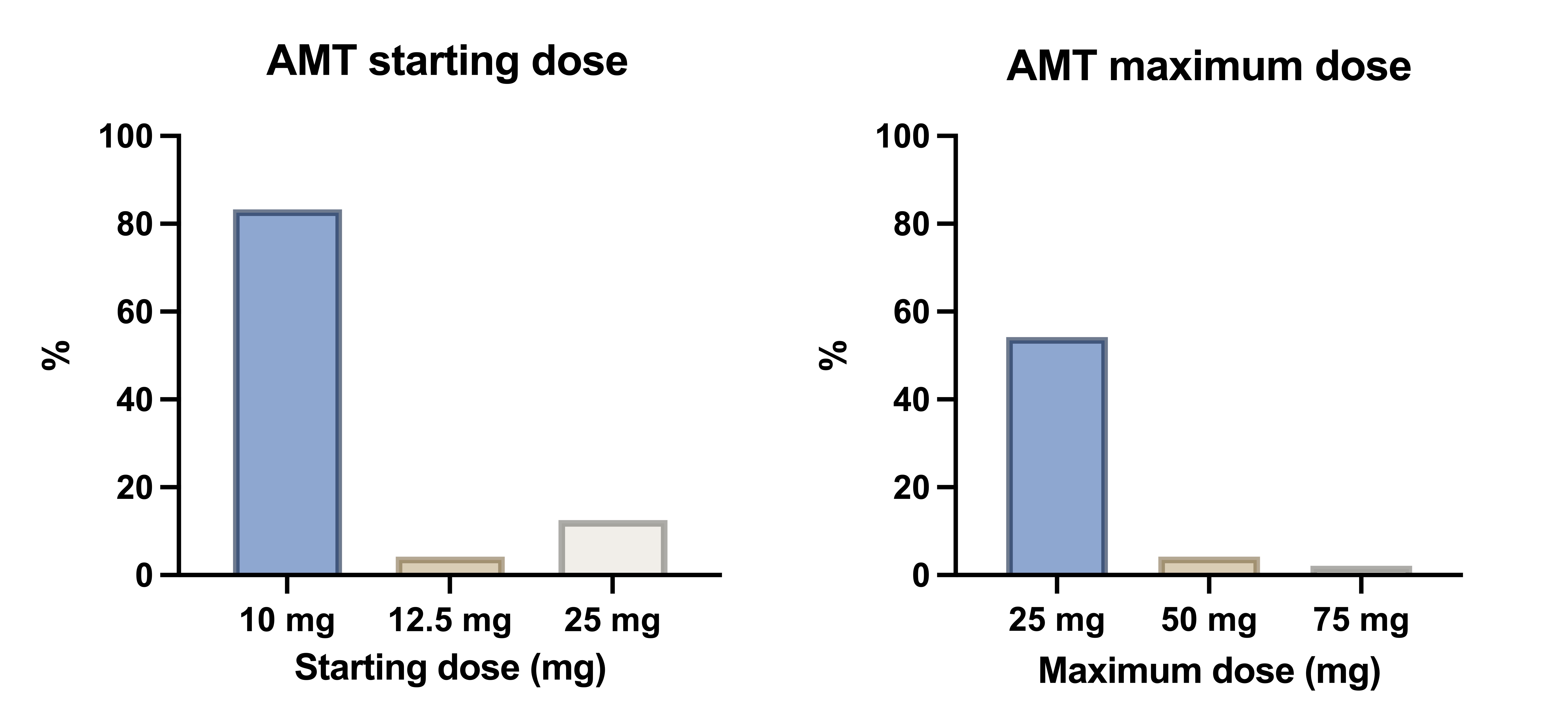

Supplement: Supplementary file 2 — Supplementary file2 (JPG 672 KB) [file 415_2022_11225_MOESM2_ESM.jpg]
