# Supplementary material for: Amitriptyline for post-COVID headache: effectiveness, tolerability, and response predictors
Source: J Neurol. 2022 Jul 12;269(11):5702–9. doi: 10.1007/s00415-022-11225-5 (PMC9553757; doi:10.1007/s00415-022-11225-5)
Supplement: Supplementary file 3 — Supplementary file3 (DOCX 21 KB) [file 415_2022_11225_MOESM3_ESM.docx]

**Table S1. Univariate and multivariate regression analysis.** All variables were included in the univariate linear regression analysis; variables with a p value under 0.1 were subsequently included in the multivariate analysis.

| Variable | Analysis | B value | 95% CI lower limit, CI upper limit | p value |
| --- | --- | --- | --- | --- |
| Sex | Univariate | -0.400 | (-9.047, 8.247) | 0.926 |
|  | Multivariate |  |  |  |
| Age, years | Univariate | -0.051 | (-0.290, 0.189) | 0.672 |
|  | Multivariate |  |  |  |
| History of migraine | Univariate | 1.842 | (-5.089, 8.774) | 0.595 |
|  | Multivariate |  |  |  |
| History of TTH | **Univariate** | **11.535** | **(1.555, 21.515)** | **0.024*** |
|  | **Multivariate** | **10.226** | **(0.265, 20.188)** | **0.044*** |
| Comorbid anxiety or depression | **Univariate** | **7.862** | **(0.558, 15.167)** | **0.035*** |
|  | Multivariate | 3.862 | (-3.5, 11.223) | 0.296 |
| Comorbid insomnia | Univariate | 7.037 | (-0.342, 14.415) | 0.061 |
|  | Multivariate | 1.521 | (-6.108, 9.150) | 0.689 |
| Other concomitant pain | Univariate | 2.063 | (-7.049, 11.174) | 0.651 |
|  | Multivariate |  |  |  |
| Prior number of prophylactics for post-COVID-19 headache | Univariate | -0.268 | (-5.271, 4.735) | 0.915 |
|  | Multivariate |  |  |  |
| Intensity (VAS) | Univariate | 0.243 | (-1.296, 1.782) | 0.752 |
|  | Multivariate |  |  |  |
| Hemicranial | Univariate | -4.632 | (-12.448, 3.185) | 0.239 |
|  | Multivariate |  |  |  |
| Holocranial | Univariate | 1.474 | (-6.450, 9.398) | 0.710 |
|  | Multivariate |  |  |  |
| Oppressive | Univariate | 1.545 | (-10.107, 13.198) | 0.791 |
|  | Multivariate |  |  |  |
| Throbbing | Univariate | -4.444 | (-11.770, 2.881) | 0.228 |
|  | Multivariate |  |  |  |
| Nausea | **Univariate** | **-9.531** | **(-15.756, -3.307)** | **0.003*** |
|  | **Multivariate** | **-8.594** | **(-14.717, -2.470)** | **0.007**** |
| Vomiting | Univariate | -6.286 | (-15.851, 3.279) | 0.192 |
|  | Multivariate |  |  |  |
| Photophobia | Univariate | -1.938 | (-8.750, 4.875) | 0.570 |
|  | Multivariate |  |  |  |
| Phonophobia | Univariate | -4.469 | (-11,176, 2.238) | 0.186 |
|  | Multivariate |  |  |  |
| Osmophobia | Univariate | -10.493 | (-23.683, 2.698) | 0.116 |
|  | Multivariate |  |  |  |
| Allodynia | Univariate | -2.993 | (-12.658, 6.673) | 0.536 |
|  | Multivariate |  |  |  |
| Worsening with headache movement | Univariate | -2.447 | (-8.906, 4.012) | 0.449 |
|  | Multivariate |  |  |  |
| Baseline number of headaches per month (days) | Univariate | 0.352 | (-0.173, 0.878) | 0.183 |
|  | Multivariate |  |  |  |
| Baseline number of moderate-severe days per month (days) | Univariate | 0.154 | (-0.183, 0.491) | 0.363 |
| Baseline number of NSAIDs per month (days) | Univariate | 0.104 | (-0.210, 0.418) | 0.510 |
|  | Multivariate |  |  |  |
| Baseline number of triptans per month (days) | Univariate | -1.119 | (-3.173, -3.173) | 0.278 |
|  | Multivariate |  |  |  |
| Time from COVID-19 to AMT (months) | **Univariate** | **-0.641** | **(-1.402, -0.042)** | **0.036*** |
|  | Multivariate | -0.429 | (-1.253, 0.395) | 0.299 |
| Amitriptyline starting dose (mg) | **Univariate** | **-0.633** | **(-1.257, -0.009)** | **0.047*** |
|  | Multivariate | -0.322 | (-0.909, 0.265) | 0.275 |
| Maximum doses of Amitriptyline (mg) | Univariate | -0.069 | (-0.349, 0.210) | 0.621 |
|  | Multivariate |  |  |  |
| Time lapsed before doubling the starting dose (weeks) | Univariate | 0.309 | (-0.287, 0.906) | 0.302 |
|  | Multivariate |  |  |  |
| Amitriptyline duration (months) | Univariate | 0.292 | (-0.845, 1.429) | 0.607 |
|  | Multivariate |  |  |  |
| NSAIDs as symptomatic treatment | Univariate | -1.681 | (-8.754, 5.393) | 0.635 |
|  | Multivariate |  |  |  |
| Triptans as symptomatic treatment | Univariate | -9.182 | (-20.520, 2.156) | 0.110 |
|  | Multivariate |  |  |  |
| Adverse effects | Univariate | -4.340 | (-11.147, 2.467) | 0.205 |
|  | Multivariate |  |  |  |

TTH: Tension-type headache; CI: confidence interval; *p<0.05; **p<0.01
